# Surgical treatment of traumatic eventration with polyester button and polypropylene mesh to strengthen the suture technique in equine

**DOI:** 10.1186/s12917-016-0686-8

**Published:** 2016-03-19

**Authors:** Carla Faria Orlandini, Denis Steiner, André Giarola Boscarato, Gabriel Coelho Gimenes, Luiz Romulo Alberton

**Affiliations:** Department of Veterinary Medicine and Graduate Program in Animal Science, Universidade Paranaense, Praça Mascarenhas de Moraes 4282, Zona III, 87502-210 Umuarama, Paraná Brazil

**Keywords:** Abdominal laceration, Equine, Eventration, Hernia, Hernioplasty, Surgery

## Abstract

**Background:**

Defects in the abdominal wall of horses have high relapse rate. This is mainly in lateral eventrations and hernias caused by trauma from kicks of other horses or installation structures. The eventration region normally becomes swollen and there may be complications due to intestinal loop incarceration. The surgical treatment, consisting of reconstruction of the abdominal wall, frequently require biological or synthetic materials for the reinforcement of the suture line and tension support. Therefore, several studies have reported new materials for the repair of the abdominal wall, with the aim of improving the integration among adjacent tissues and reducing risks and complications such as rejection and infection. This report describes for the first time the use of a regular polypropylene mesh reinforced with polyester buttons for the herniorrhaphy.

**Case presentation:**

A male, three-year-old, Appaloosa with 500 Kg presented to our hospital with a 10 days history of an increased volume on the left ventro-lateral region of the abdomen. During the physical examination, a deventration following traumatic rupture of the abdominal wall was diagnosed via ultrasonography. Then, the equine was anesthetized and moved to surgery for correction of the eventration which was performed according to conventional technique described in literature. Two days later, an eventration relapse was observed and confirmed via ultrasonography. After that, a second surgical intervention was performed using polyester buttons and polypropylene mesh. After the second surgical procedure, no complications related to eventration were observed either intra or postoperatively. After that, a recheck was performed thirty days later where satisfactory wound healing and total recovery were observed.

**Conclusion:**

The use of polypropylene mesh reinforced with polyester buttons is an effective technique for the repair of traumatic eventration in horses. This technique provides effective reinforcement against the abdominal tension and was a good option for reconstruction of lacerated muscles in cases of equine post-traumatic eventration, including relapsing cases.

## Background

The traumatic rupture of the abdominal wall, extending to one or more muscles, predispose to the escape of viscera that, when retained by the subcutaneous tissue and skin, form the so-called eventration, or lateral abdominal hernia [1, 2]. In horses, these are usually the result of trauma, associated with facility problems where the animal is maintained and trauma caused by kicks from other animals [2]. Eventrations are classified as partially or completely reducible and usually have slow or blunt onset. The opening region normally becomes swollen and some problems may occur due to retention or definitive incarceration of abdominal viscera [1]. Surgical treatment is effective for the reduction of the eventration content and reconstruction of the abdominal wall. This can be complex, mainly when there are alterations in the anatomic composition, adherences of structures and thin friable tissue. Moreover, the weight of the abdominal viscera on the peritoneal wall is an aggravating factor, predisposing the occurrence of relapse of the eventration, requiring the reinforcement of the suture line and, many times, the use of materials to enhance tension support [2].

Several synthetic or biological materials have been applied for the reconstitution of the abdominal wall, serving as reinforcement to tension or even to stimulate scarring and the tissue regeneration processes [3, 4]. However, these materials have been associated with severe adverse reactions such as infections and rejection [5]. Synthetic prosthesis have been commonly used since 1950, being divided into non absorbable, such as nylon, polytetrafluorethylene, silicone, polyester, polypropylene, polyvinyl sponge, and carbon fibers; and absorbable, such as polygalactin 910 and polyglycolic acid [5, 6]. More recently, biological materials have been used for the same purpose. Among these, swine small bowel submucosa [7, 8], swine urinary bladder submucosa [9], bovine peritoneum [10], microbial cellulose membrane [11], and bovine pericardium [4, 12] stand out. Collagen-based animal tissue has been more frequently used, associated to several conservation techniques to preserve its viability and reduce antigenicity [13].

New materials used for abdominal wall repair have been investigated and are associated with acceptable integration with adjacent tissues, improving performance in all interfaces and preventing complications. In this context, the aim of the present paper was to report a herniorraphy using polyester buttons to enforce a polypropylene mesh in a horse with eventration secondary to traumatic rupture of the abdominal muscles.

## Case presentation

**Fig. 1:**
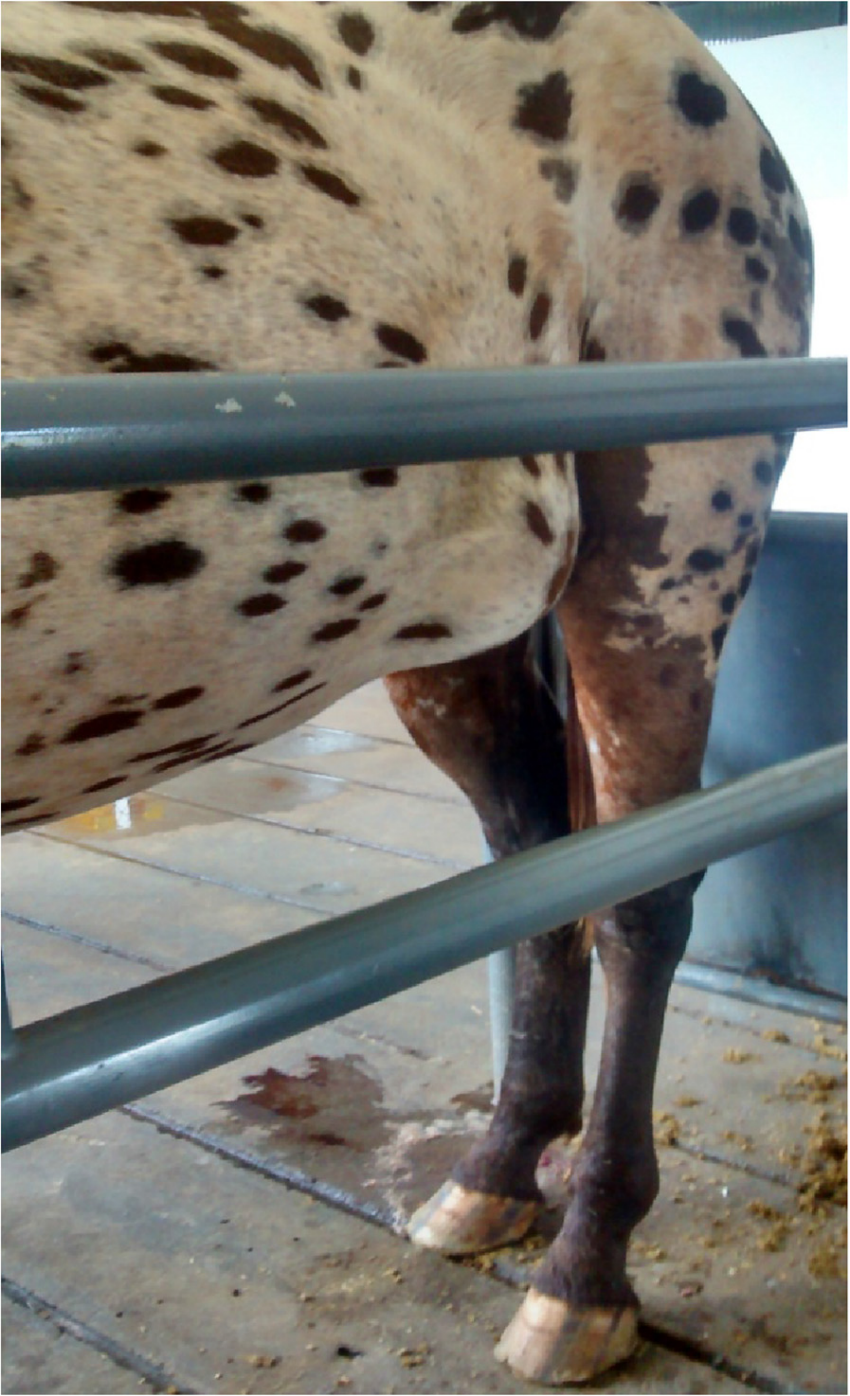
Photograph demonstrating the equine presenting increased volume on the left ventral-lateral region due to trauma

A male three-year-old, Appaloosa was admitted to the Universidade Paranaense, Veterinary Hospital presenting with a 10 days history of an increased volume of the left ventral-lateral region of the abdomen (Fig. 1).

**Fig. 2:**
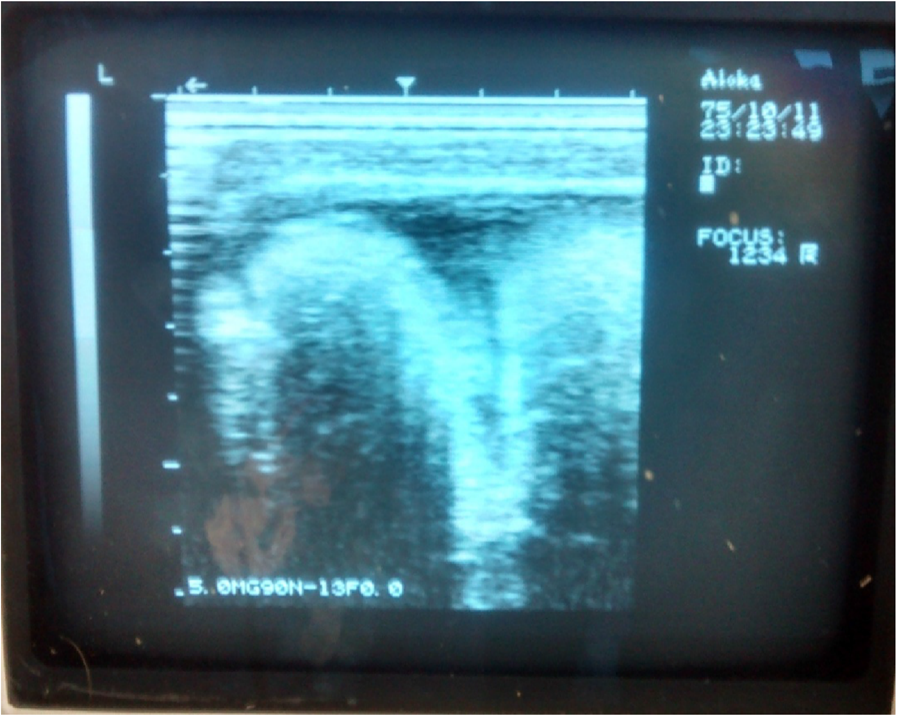
Photograph illustrating an ultrassonography demonstrating the presence of intestinal loops in the subcutaneous region in the left ventral-lateral region of the abdomen of a horse with traumatic eventration

Anamnesis showed injury due to trauma at the farm fence. The animal had already been treated with procaine benzylpenicillin (10.000 UI/Kg, IM, q24h), dexamethasone (1 mg/kg, IV, q24h) during four days and antitetanus serum. During physical examination, an increased volume with firm consistency, reducible and painful to touch was observed. Through ultrasonography, the presence of intestinal loops in the lesion area (Fig. 2) and peritoneal fluid and fibrin were observed, which confirmed the diagnostic of eventration. The animal was conducted to surgery on the day after admission.

**Fig. 3:**
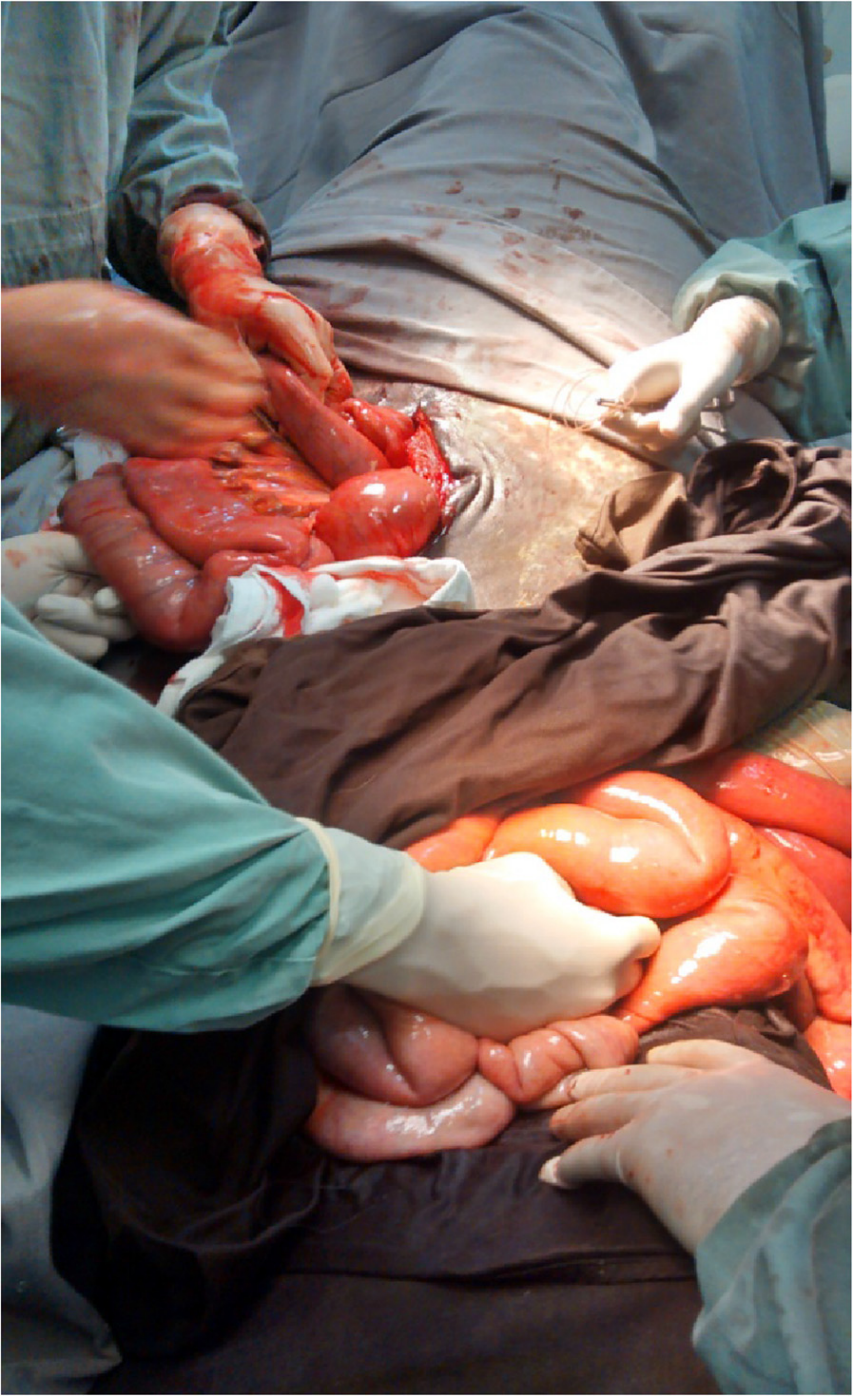
Photograph illustrating a horse undergoing surgery for treatment of traumatic eventration, showing the passage of intestinal loops through lacerations of the abdominal muscles on the left ventral-abdominal region, and incision through the retro-umbilical region for traction and repositioning of eventrated tissues

Pre surgical preparation comprised of 8-h fasting of water and 12-h fasting for solids. Anesthesia was performed with xylazine 10 % (1 mg/kg, IV), ketamine (2 mg/kg, IV), and guaiacolglyceryl ether (50 g), diluted in 0.9 % saline solution (500 ml), all administered IV as pre anesthetic medication and also for induction of general anesthesia. After oro-tracheal intubation anesthesia was maintained with isoflurane vaporized in oxygen and animal was positioned in dorsal recumbency for the surgical procedure. Then, hair clipping and preoperative antisepsis on the ventral and left ventral-lateral regions of the abdomen were performed. An incision of approximately 15-cm was performed, comprising skin and subcutaneous tissue, on the eventration region, through which it was possible to observe lacerations of the obliquusabdominis and transverses abdominus muscles, allowing the passage of intestinal loops to the subcutaneous space. To reduce the eventrated content, another incision, of approximately 20-cm was performed in the retro-umbilical region, comprising skin, subcutaneous, línea alba of the rectus abdominis and peritoneum (Fig. 3). As the lesion was located on the left ventrolateral region, the lateral position did not favor its manipulation and correction, hindering access and technique execution. In addition, the laceration of the abdominal muscles and the way the intestinal loops were eventrated impaired their anatomical replacement, with the need for a second access to aid this technique. It is noteworthy that due to the degree of muscle laceration and the time elapsed since trauma to the moment the animal was treated, the muscles abdominal oblique and transverse were extremely friable, hindering the approach of its edges, and hence difficulty to the suture of these tissues to close the laceration. Thus, the option of increasing the incision in such musculature to facilitate the repositioning of the bowel was ruled out, since this technique would make muscle reconstitution even more difficult.

After the repositioning of the intestinal loops, the peritoneum was closed in both openings of the abdominal region, using chromed catgut-4 suture line in a simple continuous pattern. Subsequently, the lacerated muscles were repaired using nylon-4 and an anchored simple continuous pattern (Reverdin suture). For the suture of the rectus abdominis muscle, nylon-4 suture material was used in a Sultan suture pattern. The subcutaneous tissue was closed with vycril-1 in a simple continuous suture patern. Skin incisions were sutured with nylon-3 in a simple interrupted pattern.

**Fig. 4:**
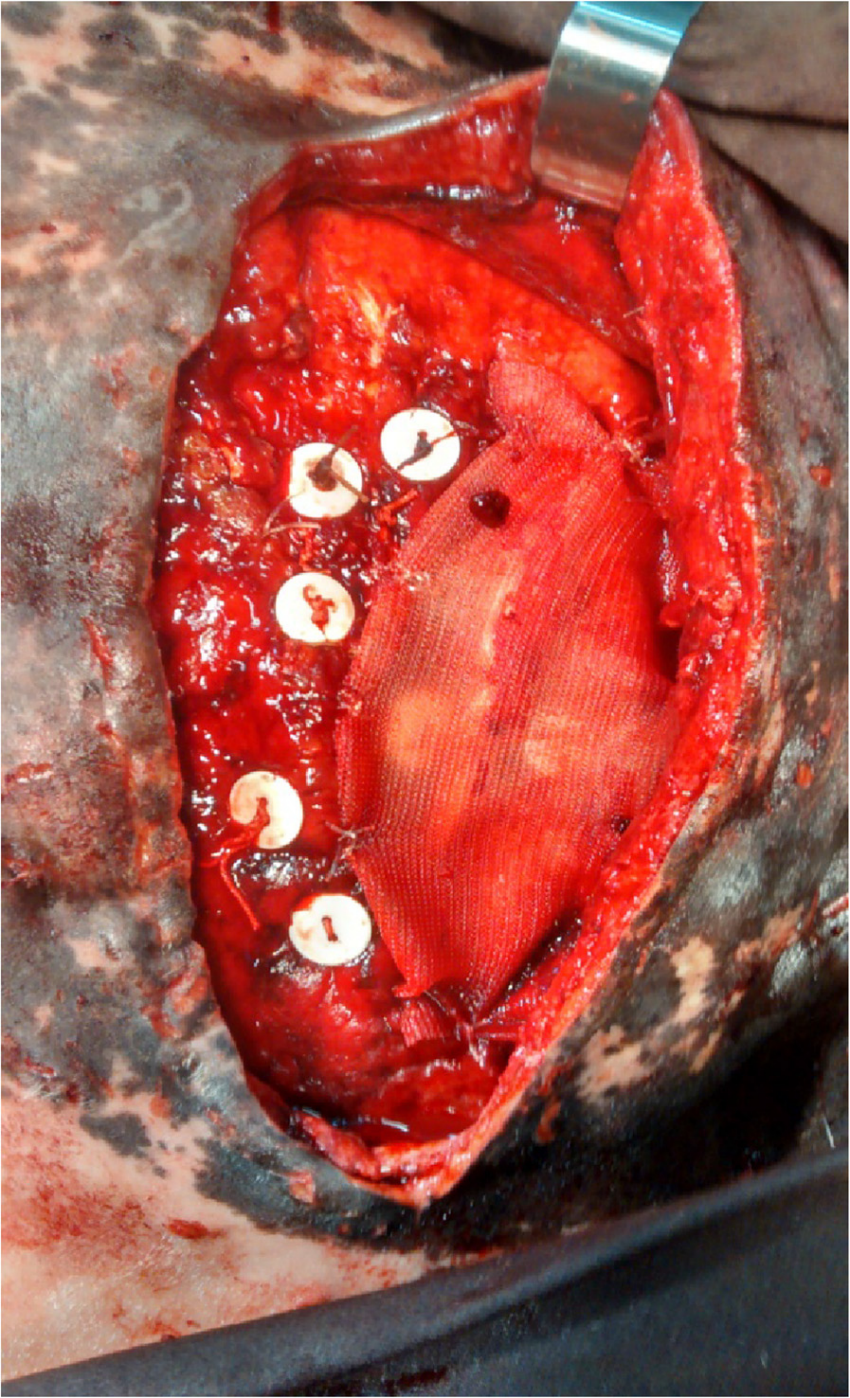
Photograph illustrating a horse undergoing surgery for treatment of traumatic eventration demonstrating the synthetic materials, polyester buttons and polypropylen mesh used for reconstruction of the obliquus and transverse abdominis

Post-surgical treatment was performed by applying cold water on the suture regions and prepuce, cleaning of the surgical wounds with iodine povidone (PVPI), PVPI solution and anointment, and administration of flunixin meglumine (1.1 mg/kg, IV, q24h) and trimethoprim-sulfamethoxazole (15 mg/kg, IV, q12h). Two days after the procedure, eventration relapse was observed and confirmed by ultrasonography. Sodium heparin (30 UI/kg, SC, q24h) was administered for two days, associated to other postoperative medication to prevent possible adherence of eventrated intestinal loops. Four days after the first surgery, a second surgical intervention was performed. The preoperative procedures, anesthesia, as well as surgical access were performed as previously described. Rupture of sutures of lacerated muscles and peritoneum was observed, the latter was closed using the same procedure as in the first surgery. For the synthesis of the obliquus and transverse abdominis, ten 12.7 mm polyester buttons and a 15 x 15 cm polypropylene mesh were used, associated to simple continuous suture stitches with nylon-4 (Fig. 4).

The closure of the subcutaneous space and skin were performed using the same procedure as in the first surgery. Postoperative treatment was made with flunixin meglumine (1.1 mg/kg, IV, q24h), for three days, ceftiofur (2.2 mg/kg, IV, q24h) and gentamicin (6.6 mg/kg, IV, q24h) during 15 days. Three days after the second surgical procedure, another dehiscence of the cutaneous suture was observed. However, with daily cleanings of the wound as previously described, application of frozen total plasma, obtained from the animal, and compressive abdominal bandages, satisfactory wound healing and total recovery were observed within 30 days.

The first surgical intervention was performed 11 days after animal had suffered the trauma and thus presenting with eventration. During this period, probably there was not enough time for strong collagen formation to support the suture performed [14], which may have contributed to the lesion recurrence, with suture rupture of lacerated muscles and peritoneum. Nevertheless, surgical procedure was performed as soon as the animal was admitted, since the presence of peritoneal fluid and fibrin, visualized through ultrasonography, indicated possible adhesion formation and hence incarceration of eventrated bowel loops, which may have contribute to the onset of acute abdomen and possible further complications. Some authors have reported surgical techniques suitable for ventral hernia repair in horses including the attempt of primary defect closure, without the use of implants [15]. In this case, primary defect closure failure due to the size of the laceration and the animal’s size [15]. The materials used to reinforce the abdominal suture were easy to apply, had good interaction with the suture and excellent resistance to traction, even when inserted in heavily lacerated muscle due to trauma and to surgical procedures. Moreover, materials were easy to acquire and had low cost. Similar results were described by using bovine pericardium preserved in 98 % glycerine for reinforcement of the peritoneal suture in horses with traumatic eventration [4]. According to authors, glycerin has low cost and is easily acquired and stored. Nevertheless, the conservation of biological membranes used as implants may be more demanding than simple sterilization used for the antisepsis of synthetic materials.

In a study comparing the use of bovine pericardium and polyester mesh for the repair of abdominal wall in rats, the authors concluded that the synthetic material used offered greater structural resistance and fibroblastic response; however, it showed higher adherence of abdominal viscera, when compared to bovine pericardium [16]. The implant of synthetic prosthesis significantly stimulates the inflammatory response [16, 17]. The results confirm the hypothesis that biomaterials induce less inflammatory response [18]. The authors used hemicellulose membrane for reconstitution of defects in the abdominal wall induced in rats and found satisfactory results. Once the biomaterial establish a certain balance with adjacent tissues, minimal inflammatory response and few intestinal adherences are observed. These responses are desirable characteristics in any kind of implant. Comparing the use of synthetic material and biomaterial for the reconstruction of abdominal defects in rats, there is no histological difference between materials in relation to inflammatory process, classified as granulomatous regardless of material used [19]. Defects in the abdominal wall in equines, corrected only by suture, present great chance of relapse and, in such cases, polypropylene meshes are a good option for reconstruction and resistance of the abdominal wall [17]. However, despite the risk of complications and relapses, most hernia or eventration lesions in large animals are corrected with the use of sutures, various techniques and wires. In cattle, some authors have reported no significant difference between synthetic nonabsorbable sutures (nylon) and organic nonabsorbable sutures (cotton) for the suturing of muscles in animals with umbilical hernia [20]. However, the authors also reported that suture with cotton wire, provides higher recovery rate when compared to nylon, arguing that the texture of organic wire is not able to fray abdominal fascia, although suture has exerted pressure on tissues. However, it should be taken into consideration that due to its physical and chemical characteristics, this type of wire may trigger greater number of complications and predispose to possible contamination. According to some authors, the failure of abdominal suture techniques in cases of hernia and the high recurrence rate are due to the individual response of animals, postoperative failure and suture patterns [21]. The suture patterns chosen for the synthesis of lacerated muscle, in the case described here were based on the fact that a tension relaxation suture cannot be used due to the difficulty in anchoring the suture with the muscle fiber. The anchored continuous suture (Reverdin suture) allowed greater sealing of the laceration site, since the infiltration of peritoneal fluid could predispose to contamination and, consequently, point dehiscence. The Sultan suture technique provided stability to the incision line, since it is supported by four points, and lower chance of muscle laceration. The occurrence of hernia recurrence after the first surgery is probably related to the weakness presented by the lacerated muscles, with high friability and inflammation.

In the present case report, buttons were used to provide resistance to laceration. However, a polypropylene mesh in combination with buttons was chosen to reinforce the suture line. The tearing and rupture of muscles were associated with higher predisposition of further abdominal defect; this required greater approximation in the wound. According to the same authors, the technique used ensures good healing for abdominal reconstruction and good recovery of patients, which can return to normal activities, requiring approximately 60 days of movement restriction. However, in the case reported here, the animal returned to its normal routine 30 days after the procedure.

## Conclusion

The use polyester buttons and polypropylene mesh, provided effective reinforcement to the abdominal tension and was deemed an effective option for reconstruction of lacerated muscles after post-traumatic eventration in a horse.

## Consent

The surgical procedure was performed after the agreement of the horse owner.

## References

[1] Knottenbelt DC, Pascoe RR (1998). Afecções e distúrbios do cavalo.

[2] Auer JA, Stick JA (2012). Equine surgery.

[3] Vulcani VAS, Macoris DG, Plepis AMG (2009). Biomateriais para reparação cirúrgica da parede abdominal emanimais domésticos revisão. Arq Ciênc Vet Zool.

[4] Stelmann UJP, Silva AA, Souza BG,Hess TM, Aguiar GC, Santos AE. Utilização de pericárdio bovino como reforço da ráfia do peritônio no tratamento cirúrgico de eventração em equino: relato de caso. Rev. Cient. Elet. Med. Vet.2010. http://faef.revista.inf.br/imagens_arquivos/arquivos_destaque/deUhbGJWXS4U9Ib_2013-6-25-15-5-8.pdf. Accessed 23 Sept 2014.

[5] Bellón JM (2005). Propuesta de una nuevaclasificación de prótesis destinadas a La reparación de defectosherniarios em lapared abdominal. CirEspan.

[6] BaergJ KG, TonitaJ PP, Reid D (2003). Gastroschisis: a sixteen-year review. JPediatrSurg.

[7] BadylakS KK, Tullius B, Simmons-ByrdA MR (2002). Morphologic study of small intestinal submucosa as a body wall repair device. JSurg Res.

[8] Greca FH, Souza Filho ZAS, Rocha SL, Borsato KS, Fernandes HAD, Niiside MA (2004). Submucosa de intestino delgado no reparo de defeito em parede abdominal de ratos. ActaCirBras.

[9] Soiderer EE, Lantz GC, Kazacos EA, Hodde JP, Wiegand RE (2004). Morphologic study of three collagen materials for body wall repair. JSurg Res.

[10] Bastos ELS, Fagundes DJ, Taha MO, Novo NF, Silvado RAB (2005). Peritônio bovino conservado na correção de hérnia ventral em ratos: uma alternativa para tela cirúrgica biológica. Rev Col Bras Cir.

[11] Falcão SC, Coelho ARB, EvêncioNeto J (2008). Biomechanical evaluation of microbial cellulose (Zoogloeasp.) and expanded polytetrafluoroethylenemembranesas implants in repair of produced abdominal wall defectsin rats. Acta CirBras.

[12] Yamatogi RS, RahalSC GJM, TagaR CTM, Lima AFM (2005). Histologia da associação de membranas biológicas de origembovina implantadas no tecido subcutâneo de ratos. Cienc Rural.

[13] VulcaniVAS MDG, Plepis AMG, Martins VCA, Franzo VS, RabeloRE S’a FJF (2013). Implantação de biomembrana de colágeno tratada em solução alcalina ou conservada em glicerina a 98 % na parede abdominal de equinos. Cienc Rural.

[14] Smeak DD (1989). Management and prevention of surgical complications associated with small animal abdominal herniorraphy. Probl Vet Med.

[15] Haupt J, García-Lopez JM, Chope K (2015). Use of a novel silk mesh for ventral midline hernioplasty in a mare. BMC Vet Res.

[16] Quitzan JG, Rahal DC, RochaNS CAJ (2003). Comparação entre pericárdio bovino preservado em glicerina e malha de poliéster no reparo de falhas da parede abdominal em ratos. Acta Cir Bras.

[17] Ober C, Muste A, Mates N, Oana L, Beteg F, Vancea C (2007). Techniques of implant of prosthetic meshes of polypropylene in repair of the abdominal wall defects in horses. Bull UnivAgric Sci Vet Med Cluj Napoca.

[18] Andrade FAG, Cavalcanti CEO, Mota PKV, Plech R, Ferreira LM (2011). Hemicelulose em reconstrução da parede abdominal em ratos. Rev Bras CirPlást.

[19] Paulo NML, Lima FG, Siqueira Júnior JT, FleuryLFF S’a FJF, Borges AC, Telles TC (2005). Membrana de látex da seringueira (*Hevea brasiliensis*), com e sem polilisina a 0,1 % e tela de marlex na reconstrução de defeitos iatrogênicos da parede abdominal de ratos. Acta CirBras.

[20] Silva LAF, Eurides D, Souza LA, Oliveira BJA, Helou JB, Fonseca AM, Cardoso LL, Freitas SLR (2012). Tratamento de hérnia umbilical em bovinos. Rev Ceres.

[21] Silva LAF, Neto JBP, Chiquetto CE, Fioravanti MCS, Eurides D, Borges GV, Atayde IB, Rabelo RE (2000). Herniorrafia umbilical em bovinos e avaliação do pós-operatório. Ciênc Anim Bras.

